# ATACseqQC: a Bioconductor package for post-alignment quality assessment of ATAC-seq data

**DOI:** 10.1186/s12864-018-4559-3

**Published:** 2018-03-01

**Authors:** Jianhong Ou, Haibo Liu, Jun Yu, Michelle A. Kelliher, Lucio H. Castilla, Nathan D. Lawson, Lihua Julie Zhu

**Affiliations:** 1 Department of Cell Biology, Duke University Medical Center, Durham, NC 27710 USA; 2 Department of Molecular, Cell and Cancer Biology, University of Massachusetts Medical School, 364 Plantation Street, Worcester, MA 01605 USA; 3 Department of Molecular Medicine, Program in Bioinformatics and Integrative Biology, Worcester, MA 01655 USA

**Keywords:** ATAC-seq, Quality control, ATACseqQC, Chromatin accessibility

## Abstract

**Background:**

ATAC-seq (Assays for Transposase-Accessible Chromatin using sequencing) is a recently developed technique for genome-wide analysis of chromatin accessibility. Compared to earlier methods for assaying chromatin accessibility, ATAC-seq is faster and easier to perform, does not require cross-linking, has higher signal to noise ratio, and can be performed on small cell numbers. However, to ensure a successful ATAC-seq experiment, step-by-step quality assurance processes, including both wet lab quality control and in silico quality assessment, are essential. While several tools have been developed or adopted for assessing read quality, identifying nucleosome occupancy and accessible regions from ATAC-seq data, none of the tools provide a comprehensive set of functionalities for preprocessing and quality assessment of aligned ATAC-seq datasets.

**Results:**

We have developed a Bioconductor package, *ATACseqQC*, for easily generating various diagnostic plots to help researchers quickly assess the quality of their ATAC-seq data. In addition, this package contains functions to preprocess aligned ATAC-seq data for subsequent peak calling. Here we demonstrate the utilities of our package using 25 publicly available ATAC-seq datasets from four studies. We also provide guidelines on what the diagnostic plots should look like for an ideal ATAC-seq dataset.

**Conclusions:**

This software package has been used successfully for preprocessing and assessing several in-house and public ATAC-seq datasets. Diagnostic plots generated by this package will facilitate the quality assessment of ATAC-seq data, and help researchers to evaluate their own ATAC-seq experiments as well as select high-quality ATAC-seq datasets from public repositories such as GEO to avoid generating hypotheses or drawing conclusions from low-quality ATAC-seq experiments. The software, source code, and documentation are freely available as a Bioconductor package at https://bioconductor.org/packages/release/bioc/html/ATACseqQC.html.

**Electronic supplementary material:**

The online version of this article (10.1186/s12864-018-4559-3) contains supplementary material, which is available to authorized users.

## Background

In eukaryotes, nuclear DNA is primarily found packaged in nucleosomes, each of which consists of ~ 147 bp of DNA coiled around a histone octamer core. Two adjacent nucleosomes are usually spaced by linker DNA of ~ 20–90 bp which can be bound by a linker histone H1 [1] . In general, the interplay between histones and DNA serves as an important regulatory point for controlling gene expression. Most notably, transcriptionally active elements, such as promoters and enhancers, are defined by short regions of DNA that are devoid of direct histone interactions. These regions of “open” chromatin are usually occupied by transcription factors that facilitate gene transcription. By contrast, the promoters of genes that are not actively expressed in a given cell type exhibit much tighter association with histones, which prevents transcription factors from activating transcription and contributes to gene repression. Given the strong correlation between open chromatin and active regulatory elements, this topological feature has become a valuable marker that researchers can use to identify putative promoter and enhancer elements of interest.

In recent years, several high-throughput methods have been developed to assess chromatin accessibility, nucleosome positioning, and occupancy of DNA-associated proteins. These include three direct chromatin accessibility methods (DNase-seq [2], FAIRE-seq [3], and ATAC-seq [4]) and one indirect method (MNase-seq [5]). Among these methods, ATAC-seq has gained considerable popularity for several reasons [6]. First, chromatin accessibility profiles identified by ATAC-seq are comparable to other methods, including MNase-seq, DNase-seq and FAIRE-seq, while yielding comparable or even higher signal-to-noise ratios [7, 8]. Second, ATAC-seq is easier and faster to carry out than other methods for assaying chromatin accessibility. Third, it does not require fixation of cells, thereby capturing native chromatin states. Importantly, ATAC-Seq can be reliably applied to low numbers of cells and has been successfully applied to single cells [9, 10]. More recently, ATAC-seq has been further optimized to profile chromatin states in properly frozen cells [11, 12], making the method applicable to clinical studies.

Although it is relatively easy to perform ATAC-seq experiments, analysis of ATAC-seq data is not trivial. A number of tools have been developed or adopted for assessing sequencing quality (e.g. FASTQC [13], ATAC-Seq/DNase-Seq Pipeline [14], I-ATAC [15], and ataqv [16]), identifying nucleosome occupancy (e.g. NucleoATAC [17] and DANPOS2 [18]), and accessible chromatin regions, a.k.a. peaks (MACS2 [19]). However, none of the tools provide a comprehensive set of functionalities for preprocessing and quality assessment of aligned ATAC-seq datasets. For example, ATAqC/ATAC-seq/DNase-seq pipelines have been used for the ENCODE project, which adopt the same quality assessment (QA) criteria as ChIP-seq data such as the use of duplication metrics, but do not implement any ATAC-seq-specific QA. To help researchers preprocess and quickly evaluate the quality of their ATAC-seq data, we developed a Bioconductor package, *ATACseqQC*. Our *ATACseqQC* package not only includes most of the commonly adopted QA metrics such as fragment size distribution, mitochondrial read fraction, duplication rate, and aggregated read distribution along proximal promoters, but also provides new functionalities such as gene-centric view of signal distribution, library complexity evaluation and sequencing depth analysis.

## Methods

### Implementation and functionalities of *ATACseqQC*

*ATACseqQC* is implemented as a Bioconductor [20] package in R [21], a popular programming language and framework for statistical computation and graphics. Main functions implemented in the *ATACseqQC* package are listed in Table 1.

**Table 1:** Functions implemented in the *ATACseqQC* package

Function Name	Usage Description
*readBamFile*	Read in bam files to R leveraging Rsamtools and create a GAlignments object
*bamQC*	Perform quality assessment on alignments and Filter BAM files to remove duplicates, mitochondrial reads and low-quality or discordant alignments
*fragSizeDist*	Plot size distribution of sequenced fragments in ATAC-seq libraries
*IGVSnapshot*	Streamline the visualization of read distribution along genomic regions of interest, such as those containing housekeeping genes
*shiftGAlignmentsList*	Shift 5′ end of aligned reads in GAlignments object
*shiftReads*	
*splitGAlignmentsByCut*	Split the shifted bam files based on ranges of fragment sizes in nucleosome-free, mono-, di-, tri-nucleosome bins and so on
*splitBam*	Shift 5′ end of aligned reads and split the updated bam files in one step
*writeListOfGAlignments*	Export lists of GAlignment objects back into bam files
*enrichedFragments*	Get enrichment signals for nucleosome-free and nucleosome-bound signals
*pwmscore*	Calculate the maximal similarity score for each given sequence against a PWM of a TF binding motif
*factorFootprints*	Discover and visualize footprints of a given transcription factor
*plotFootprints*	
*readsDupFreq*	Estimate library complexity, available for version 1.3.12 or later
*estimateLibComplexity*	
*saturationPlot*	Plot saturation curves based on the total number or width of significant peaks detected for a serial of subsamples, available for version 1.3.12 or later

To promote component reusability and compatibility among Bioconductor packages, several existing Bioconductor packages are leveraged. Alignment results in BAM files are first efficiently imported into R for quality assessment as *GenomicAlignments* objects using the *readBamFile or scanBam* function in the *Rsamtools* [22] and *GenomicRanges* [23] packages*.* The *bamQC* function implemented in our package can be used to assess the quality of the alignments and to generate filtered BAM files by removing reads with low alignment scores and reads derived from mitochondrial DNA or PCR/optical duplicates. In addition, this function outputs the percentage of reads of mitochondrial origin, duplication metrics (percentage of duplicate reads, non-redundant fraction (NRF), PCR bottleneck coefficients 1 and 2 [24]), and other mapping statistics.

Next, the *fragSizeDist* function in *ATACseqQC* can be used to plot the fragment size distributions of the filtered BAM files. Then, coordinates of read alignments are shifted using the *shiftGAlignmentsList* and *shiftReads* functions in *ATACseqQC* as described [4]. Two functions, *splitGAlignmentsByCut* and *writeListOfGAlignements*, are implemented for separating shifted reads into nucleosome-free and oligo-nucleosome-bound regions, which are used for the following analyses. To visualize aggregated signals around transcription start sites (TSSs) as heatmaps and histograms, the *enrichedFragments*, *featureAlignedHeatmap* and *matplot* functions from packages *ATACseqQC*, *ChIPpeakAnno* and *graphics* [25, 26] are used. In addition, *IGVSnapshot* is implemented to allow streamlined visualization of read distribution along any genomic regions of interest such as those containing housekeeping genes, leveraging the *SRAdb* package [27] and the Integrative Genomics Viewer (IGV) [28].

For transcription factor footprint analysis, *pwmScore*, *plotFootprints* and *factorFootprints* are implemented in *ATACseqQC*. It makes use of genomic sequences as *BSgenome* objects, available for various reference genomes, which can be efficiently accessed by methods in the *BSgenome* package [29], and of the position frequency matrices (PFMs) of binding motifs of transcription factors from the Jaspar database in the *MotifDb* package [30]. The footprint analysis also leverages the *matchPWM* function in the *BSgenome* package [29, 31] to search potential binding sites for a given DNA-binding protein, represent the matched genomic coordinates as *GenomicRanges* objects, and plot the motif as a sequence logo using the *motifStack* package [32]. The *factorFootprints* function first uses the *matchPWM* function to predict the binding sites with an input position weight matrix (PWM) for a DNA-binding protein. Next, it calculates and plots the average cutting rates for those binding sites and 100-bp flanking sequences.

For the library complexity evaluation and sequence depth analysis, *readsDupFreq*, *estimateLibComplexity*, and *saturationPlot* are implemented in *ATACseqQC*. The *estimateLibComplexity* function is built on the *ds.mincount.bootstrap* function implemented in *preseqR* [33].

An installation guide and additional generic use cases for *ATACseqQC* are described in the vignette and manual provided with the package.

Case studies.

Twenty-five ATAC-seq datasets from four studies were downloaded from NCBI SRA (Table 2) and analyzed to illustrate the utilities of *ATACseqQC* [4, 34, 35] (Vallés AJ and Izquierdo-Bouldstridge A. unpublished). First, sequence files in the SRA format were converted to the fastq format using the SRA toolkits. Then, the quality of raw reads per library was assessed using FASTQC [13]. Reads were then aligned to the human reference genome GRCh38.p10 using the aligner BWA-mem with default settings except an explicit option: -M. SAM files for read alignments were converted into sorted BAM files and filtered using SAMtools (v1.4.1) [36] to remove reads meeting the following criteria: (1) reads aligning to the mitochondrial genome; (2) reads from PCR/optical duplicates; (3) reads with mapping quality less than or equal to 20; (4) read pairs aligned discordantly; and (5) read pairs with mapping template shorter than 38 bp or greater than 2000 bp.

**Table 2:** ATAC-seq datasets used for the ATACseqQC case studies. The four datasets chosen for detailed quality control are highlighted in bold

SRA Run Accession	Condition	Comment	Study Description	Reference
SRR891269	EBV-transformed lymphoblastoid cell line	GM12878, 50 k cells	Transposition of native chromatin for fast and sensitive epigenomic profiling of open chromatin, DNA-binding proteins and nucleosome position	[4]
SRR891270	EBV-transformed lymphoblastoid cell line	GM12878, 50 k cells		
SRR891271	EBV-transformed lymphoblastoid cell line	GM12878, 50 k cells		
SRR891272	EBV-transformed lymphoblastoid cell line	GM12878, 500 cells		
SRR891274	EBV-transformed lymphoblastoid cell line	GM12878, 500 cells		
SRR891275	CD4+ T-cells purified using negative selection	CD4+ T cells, day 1		
SRR891276	CD4+ T-cells purified using negative selection	CD4+ T cells, day 1		
SRR891277	CD4+ T-cells purified using negative selection	CD4+ T cells, day 2		
SRR891278	CD4+ T-cells purified using negative selection	CD4+ T cells, day 2		
SRR3295017	Uninfected	HFF_uninfected	Toxoplasma gondii remodels the cis-regulatory landscape of infected human host cells	[30]
SRR3295018	HFF cells, uninfected	HFF_uninfected		
SRR3295019	HFF cells, uninfected	HFF_uninfected		
SRR3295020	HFF cells infected with *T. gondii*	HFF_infected		
SRR3295021	HFF cells infected with *T. gondii*	HFF_infected		
SRR3295022	HFF cells infected with *T. gondii*	HFF_infected		
SRR5720369	J-Lat A72 cells treated with DMSO	Replicate 1	The Short Isoform of BRD4 Promotes HIV-1 Latency by Engaging Repressive SWI/SNF Chromatin Remodeling Complexes	[29]
SRR5720370	J-Lat A72 cells treated with JQ1	Replicate 1		
SRR5720371	J-Lat A72 cells treated with DMSO	Replicate 2		
SRR5720372	J-Lat A72 cells treated withJQ1	Replicate 2		
SRR5800797	Breast cancer cell line T47D, multiH1sh Control	Replicate 1, 75 k cells	Analysis of the DNA accessibility upon knocking-down multiple histone H1 variants by ATAC-seq	Vallés AJ and Izquierd-Bouldstridge A., unpublished
SRR5800798	Breast cancer cell line T47D, multiH1sh Control	Replicate 2, 75 k cells		
SRR5800799	Breast cancer cell line T47D, multiH1sh Dox	Replicate 1, 75 k cells		
SRR5800800	Breast cancer cell line T47D, multiH1sh Dox	Replicate 2, 75 k cells		
SRR5800801	Breast cancer cell line T47D, RDsh control	Replicate 1, 75 k cells		
SRR5800802	Breast cancer cell line T47D, RDsh Dox Control	Replicate 1, 75 k cells		

Post-alignment quality of the ATAC-seq data was assessed by using our *ATACseqQC* package. First, we determined the fragment size distributions of the filtered BAM files. Given that size distributions of libraries from the same studies were more similar than those from different studies, further quality control steps were performed only for the representative sequencing libraries from each study using reads aligned to human chromosomes 1 and 2 unless otherwise stated. Coordinates of read alignments were shifted as described [4]. Based on the inferred size of the sequenced fragments, read alignments were split into nucleosome-free bin (38–100 bp), intermediate bin 1 (100–180 bp), mono-nucleosome bin (180–247 bp), intermediate bin 2 (247–315 bp), di-nucleosome bin (315–473 bp), intermediate bin 3 (473–558 bp), tri-nucleosome bin (558–615 bp), and others (615–2000 bp) [4]. For plotting read coverage signal around TSSs from different inferred chromatin states, reads in nucleosome-free and mono-nucleosome bins were directly used, while reads in di- and tri-nucleosome bins were extended based on their aligned templates and then were split into two and three reads, respectively. Reads in intermediate bins and longer than 615 bp were not included for plotting signal distribution around TSSs. Footprints of a DNA binding protein, CTCF, were also assessed.

Sequencing depth analysis was performed for a high quality ATAC-seq dataset (SRR891270). MACS2 was used to call broad peaks for a series of subsamples (10%, 20%, 30%, …, 80%, 90%) of the filtered BAM file and the full dataset. The number of significant peaks as well as the total width of significant peaks (FDR ≤ 0.05) from each subsample and the full dataset was plotted against the size of the corresponding subsample or the full dataset. The *loess.smooth* function from the *stats* package was used to generate a smoothed saturation curve.

Library complexities were evaluated for five ATAC-seq datasets (three 50 K–cell replicates: SRR891269-SRR891271 and two 500-cell replicates: SRR891272 and SRR891274), using the BAM files with mitochondria-derived reads removed.

To assess the effect of sequencing depth on diagnostic plots, the BAM file from a high-quality dataset (SRA run accession SRR891270) [4] was subsampled to 10%, 25%, 50% and 75% of the total number of filtered alignments. All resulting sub-datasets were assessed using *ATACseqQC*.

Scripts used for the case studies are available in Additional file 1.

## Results

To help researchers quickly assess the quality of their ATAC-seq datasets, we have implemented a comprehensive set of functionalities in *ATACseqQC*. Detailed functional comparisons between *ATACseqQC* and existing tools are listed in Additional file 2: Table S1. Below, we demonstrate the utilities of our package using 25 publicly available ATAC-seq datasets from four studies.

### Quality assessment of raw and aligned reads, and filtering alignments

For bioinformatics analysis of ATAC-seq data, quality of reads per library is assessed using FASTQC. If raw reads pass FASTQC quality control, then they can be aligned to a reference genome of choice using BWA-mem [37], Bowtie (for ≤ 50-bp reads), or Bowtie2 (for > 50-bp reads) [38]. Otherwise, quality-based trimming of reads needs to be performed using tools such as Trimmomatic [39]. Adaptor trimming is optional since these aligners can conduct soft-clipping during alignment. Before performing downstream quality assessment and other analysis, the resulting read alignments are evaluated and filtered using the *bamQC* function in the *ATACseqQC* package or external tools, such as SAMtools [36], to remove identical alignments most likely resulting from PCR/optical duplicates. In addition, reads mapping to non-nuclear (e.g. mitochondrial) DNA, which is nucleosome-free and an ideal substrate for Tn5 transposase, are removed, as are those of low mapping quality or those exhibiting discordant mapping.

Per base quality assessment results from FASTQC, and summary statistics of read mapping and filtering using BWA-mem [37] and SAMtools [36] for the 25 ATAC-seq samples are shown in Additional file 3: Table S2. The results from FASTQC show that all 25 datasets have very good sequencing quality. In contrast, the proportions of reads mapping to the mitochondrial genome showed a large degree of variation (1.2–74.0%) from study to study, although the proportions were more similar within studies, as were the sequence duplication rates (0.6–38.0%). These results underscore the importance of depleting mitochondria during nuclei preparation to make ATAC-seq more cost effective, and justify the necessity of filtering mitochondrial reads and duplicates as preprocessing steps.

### Assessment of insert size distribution

ATAC-seq leverages the hyperactive Tn5 transposase, preloaded with sequencing adaptors, to simultaneously fragment transposase-accessible DNA and tag the fragmented DNA with the sequencing adaptors, a process called tagmentation. Tn5 transposase preferentially inserts sequencing adaptors into chromatin regions of higher accessibility. Notably, besides in vivo chromatin states, frequency of Tn5 transposition also depends on DNA sequence [40] and transposase concentration. It is always recommended to optimize the ratio of cell number and enzyme concentration to better capture in vivo chromatin accessibility profiles. Thus, the size distribution of sequenced fragments of ATAC-seq libraries is an important metric of quality assessment.

**Fig. 1:**
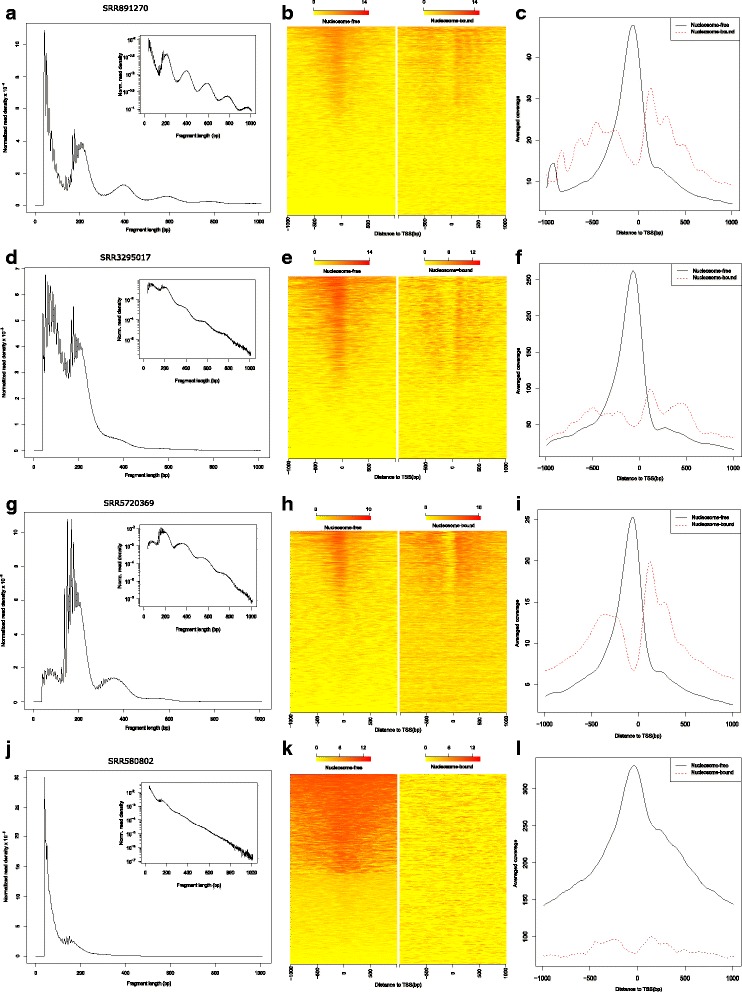
Diagnostic plots for four representative ATAC-seq datasets: SRR891270, SRR3295017, SRR5720369 and SRR580802. (**a**, **d**, **g** and **j**) Size distributions of sequenced fragments with reads passed filtering criteria for each library. (**b**, **e**, **h** and **k**) Heatmaps showing the distributions of signals around transcription start sites (TSSs), resulting from inferred nucleosome-free fragments and nucleosome-bound (mono-, di- and tri-nucleosome) fragments. To plot TSS-associated signals arising from nucleosome-bound fragments, fragments associated with di- and tri-nucleosomes were split into two and three sub-reads in silico, respectively. (**c**, **f**, **i** and **l**) Smoothed histograms of signals showing in **b**, **e**, **h** and **k**. The sample corresponding to SRR891270 was optimally transposed by Tn5, preloaded with sequencing adapters, while the sample resulting in SRR580802 was over-transposed. The other two datasets were resulted from sub-optimal transposition. Biased size selection could have occurred during library preparation for SRR5720369. Shown here are signals around TSSs on the human chromosomes 1 and 2

**Fig. 2:**
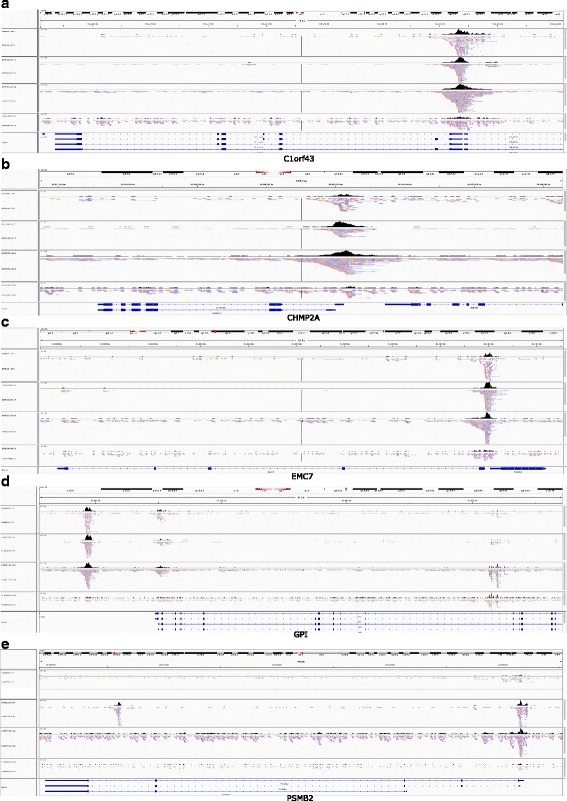
Read distribution along genomic regions containing housekeeping genes for the optimal (SRR891270), near optimal (SRR3295017 and SRR5720369) and over-transposed (SRR580802) ATAC-seq libraries. (**a**) C1orf43; (**b**) CHMP2A; (**c**) EMC7; (**d**) GPI; and (**e**) PSMB2

High quality ATAC-seq libraries generally contain about 50% of post-filtering reads as short fragments (< 100 bp), which represent nucleosome-free regions. In addition, the Tn5 transposase inserts sequencing adaptors into the linker DNA between neighboring nucleosomes. The remaining reads from larger fragments therefore come from nucleosome-bound but open chromatin regions. The insert size distribution of all the fragments should show an obvious downward laddering pattern reflecting the amount and length of DNA fragments from nucleosome-free regions, and those associated with one to several nucleosomes. The size distributions of filtered sequence fragments for all 25 samples from the four studies are plotted using *fragSizeDist* (Fig**.** 1 and Additional file 4: Figure S1). The results suggest that size distributions of libraries within studies are more similar to each other than those between studies. A typical size distribution plot for a successful ATAC-seq experiment is shown in Fig**.** 1a. Such plots can generate valuable insights into how to improve sample preparation. For example, results such as those in Fig**.** 1j suggest that ATAC-seq experiments with a too high ratio of Tn5 transposase concentration to the number of cells often leads to over-transposition, resulting in increased background signals and reduced signal-to-noise ratio (also see Fig**.** 2 and Additional file 5: Figure S2). In comparison, size distributions like those in Fig**.** 1g might have resulted from biased size selection during library preparation, likely due to an improper ratio of magnetic beads to DNA concentration [41, 42].

### Preprocessing read alignments in BAM files

The Tn5 transposase has been shown to function as a dimer and inserts the two sequencing adaptors into target regions separated by 9 bp [43]. For downstream analysis, such as peak-calling and footprint analysis, coordinates of all read alignments in the filtered BAM file thus need to be shifted. Within the *ATACseqQC* package, the function *shiftGAlignmentsList* can be used to shift the chromosomal location of the aligned reads. By default, 5′ of the reads aligned to the positive and negative strands are offset by + 4 bp and − 5 bp, respectively. Additionally, prior to drawing several other diagnostic plots, reads need to be separated into different bins based on their inferred in vivo chromatin origins, i.e., nucleosome-free and oligo-nucleosome-bound such as mono-, di-, and tri-nucleosome regions, using the function *splitGAlignmentsByCut*.

### Genome-wide and gene-centric visualization of signals around transcription start sites (TSSs)

Promoter regions of active genes are in an open chromatin state. Ideally, ATAC-seq fragments shorter than 100 bp (i.e., inferred nucleosome-free fragments) should cluster immediately upstream of TSSs. By contrast, fragments corresponding to mono-, di- or tri-nucleosomes should be depleted from TSSs of active promoters throughout the genome, but display periodic peaks of read density immediately upstream and downstream of those TSSs. Signals around TSSs from nucleosome-free fragments and from oligo-nucleosome-occupied fragments are shown as side-by-side heatmaps and the average read coverage plots for four representative ATAC-seq libraries from 4 different studies (Fig. 1b, c, e, f, h, i, k, and l). The promoters are ordered by descending signal intensities of nucleosome-free fragments at the TSSs. Side-by-side heatmaps, depicting signals around TSSs from nucleosome-free fragments and from oligo-nucleosome-occupied fragments, facilitates the visualization of expected or unexpected nucleosome patterning. Figure 1b and c show a successful ATAC-seq experiment with an increased signal immediately proximal to TSSs in nucleosome-free bins and nucleosome occupancy patterns in the further neighboring regions around TSSs in the nucleosome-bound bins. In contrast, Fig. 1k and l depict a failed experiment where there is almost no enrichment of signal around TSS from inferred nucleosome-free reads and the nucleosome positioning signals are barely detected. This could be caused by over-transposition during tagmentation as there are several-fold more nucleosome-free reads than nucleosome-bound reads (Fig. 1j and l).

While the heatmaps and histogram plots provide a genome-wide overview of aggregated signals around TSSs, read distribution along specific genomic regions, such as those containing actively transcribed genes and their flanking regions, can give an more intuitive and detailed view of the quality of ATAC-seq data. Therefore, we have developed the function *IGVSnapshot* to allow streamlined visualization of ATAC-seq results at genomic regions of interest. Housekeeping genes are known to be expressed across many tissue types [44]. Therefore, signal enrichment is expected in some regulatory regions of housekeeping genes in successful ATAC-seq experiments, which provides valuable insights into the quality of the ATAC-seq library. As expected, signals are enriched at the proximal promoters and/or enhancers of 10 human housekeeping genes, including C1orf43, CHMP2A, EMC7, GPI and PSMB2, for the optimal or near-optimal ATAC-seq libraries (SRR891270, SRR3295017 and SRR5720) (Fig**.** 2 and Additional file 5: Figure S2). By contrast, enrichment was barely observed in the over-transposed ATAC-seq library (SRR580802), even though many more reads were sequenced for this library. These results suggest that the signal distribution around housekeeping genes could serve as another indicator of library quality.

### Assessment of footprints of DNA-binding factors

**Fig. 3:**
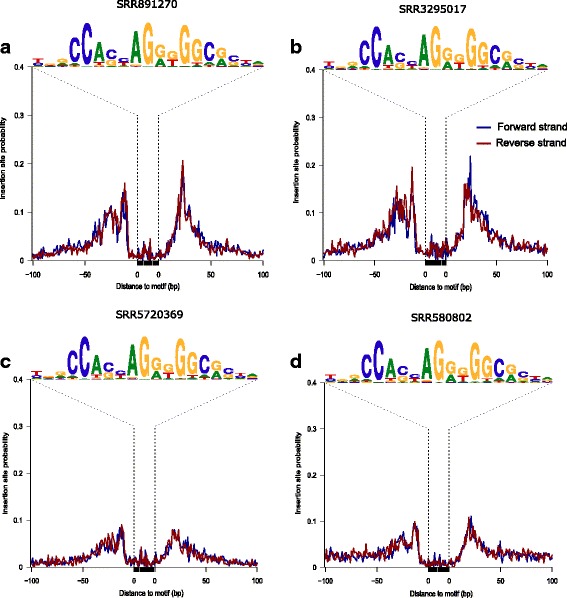
CTCF footprints inferred from the four representative ATAC-seq datasets: SRR891270 (**a**), SRR3295017 (**b**), SRR5720369 (**c**) and SRR580802 (**d**). Shown here are aggregated CTCF footprints on the human chromosomes 1 and 2

In open chromatin regions, DNA stably bound by DNA-binding proteins, such as transcription factors (TFs), can be protected from Tn5-mediated insertion of sequencing adaptors, while the flanking open regions are not. As a result, these protein-bound regions will be depleted of signal from adaptor insertions and are referred to as “footprints.” Thus, the existence of a “footprint” suggests the presence of a DNA-binding protein at that site. By plotting the aggregated signals from short-reads (< 100 bp) along predicted binding sites for DNA-binding proteins, we expect to observe “footprints” at known binding motifs. The *factorFootprints* function can be used to generate footprint plots. It first uses the *matchPWM* function in the *Bsgenome* package to predict the binding sites with an input position frequency matrix (PFM) for a DNA-binding protein. Next, it calculates and plots the average cutting rates for those binding sites and 100-bp flanking sequences. Footprints of a DNA-binding protein, CTCF, for the four representative ATAC-seq libraries are shown in Fig**.** 3. Fig**.** 3a shows clear CTCF footprints while Fig**.** 3d has a much shallower valley or less obvious footprints, despite the fact that more than two folds of reads were sequenced for the experiment corresponding to Fig**.** 3d than for the experiment corresponding to Fig**.** 3a, indicative of a non-optimized ATAC-seq experiment condition.

### Assessment of sequencing depth and library complexity

**Fig. 4:**
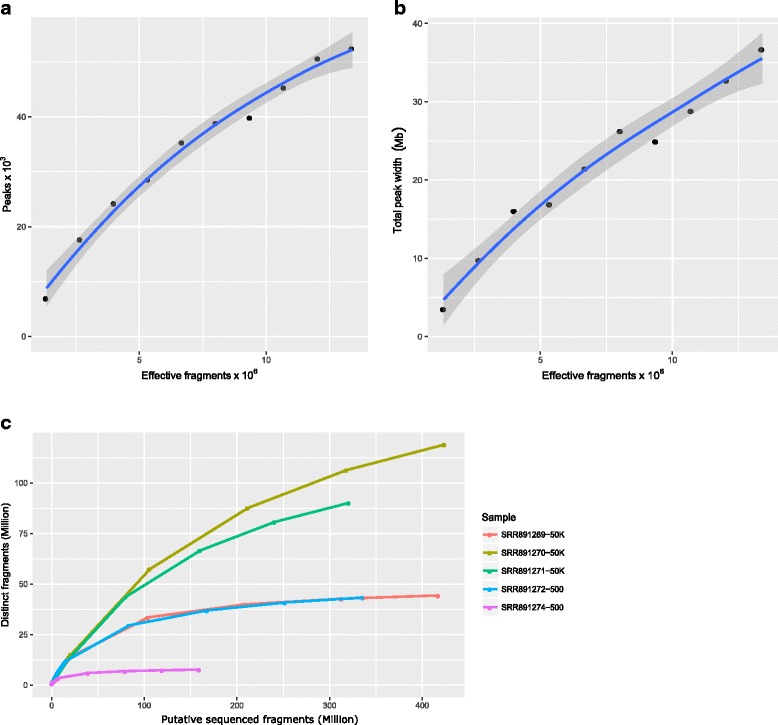
Sequence depth analysis and library complexity evaluation. It is important to know that it is not meaningful to perform saturation analysis of sequencing depth or library complexity for over-transposed ATAC-seq assays. (**a**) Total peak number-based saturation analysis of sequencing depth for SRR891270. Sequenced fragments in the filtered BAM file (called effective fragments here) are subsampled to get 10%, 20%, 30%…, 80% and 90% of total effective fragments. Broad peaks were called for each subsample and the full dataset using MACS2. The numbers of significant peaks (FDR ≤ 0.05) are plotted against the corresponding numbers of effective fragments. A smooth curve is fitted by using the *geom_loess* function in the ggplot2 package. The gray band shows the 95% confidence interval of the predicted peak numbers. (**b**) Total peak width-based saturation analysis of sequencing depth for SRR891270. The same procedure is used to fit the saturation curve except that the total width of significant peaks (FDR ≤ 0.05) for each subsample and the full dataset is used. (**c**) Library complexity analysis results for SRR891269-SRR891271, three biological replicates using 50 K cells, and for SRR891272 and SRR891274, two biological replicates using 500 cells. Number of distinct fragments was estimated for each given number of putative sequenced fragments free of mitochondrial reads

The results from both peak number- and peak width-based saturation analysis suggest that ATAC-seq library SRR89127 was not sequenced to a saturated depth, although the rate of increase in the total number or width of peaks decreases slightly after 10 million fragments passing filtering criteria (Fig**.** 4a and b). To determine whether the library is complex enough to warrant further sequencing, we performed library complexity evaluation for this library using the function *estimateLibComplexity*, along with two additional biological replicates containing 50 K cells and two libraries from 500 cells. As shown in Fig**.** 4c, the library complexities are greater for the libraries containing higher number of cells compared to those containing lower number of cells, and are different even among biological replicates. These results identified two 50 K–cell libraries with higher complexities for further sequencing. It is important to note that library complexity may not be comparable among different treatment conditions, cell types or developmental stages due to variations in chromatin states. However, biological replicates should have similar library complexity.

### Assessment of the effect of sequencing depth on some diagnostic plots

To determine whether sequencing depth affects the patterns in various diagnostic plots, we randomly subsampled the BAM files from a successful experiment (SRR891270). The results show that the fragment size distribution and the aggregated signals around TSSs from the subsamples with as low as 2.6 million uniquely mapped reads (Additional file 6: Figure S3) exhibit similar patterns to that of the full dataset (more than 26 million uniquely mapped reads) (Fig**.** 1a–c), and are easily distinguishable from that of the failed experiment (Fig**.** 1j–l). By contrast, although the footprints from the subsamples remain evident (Additional file 6: Figure S3), the height of the valley decreases as the depth decreases. These results suggest that fragment size distribution and nucleosome positioning pattern around TSSs are robust indicators of the quality of ATAC-seq data, and footprint patterns are more comparable for experiments with similar number of uniquely mapped reads, at least for stably bound DNA-binding proteins such as CTCF. In light of these observations, we recommend using a subset of the uniquely aligned reads as low as 3 million for generating all diagnostic plots except footprints to speed up the in silico quality control process.

## Discussion

ATAC-seq libraries are usually sequenced in a paired-end mode for better estimating insert size distribution and inferring in vivo chromatin states associated with the reads. In addition to the multiple steps of quality control performed before ATAC-seq libraries are sequenced [9, 11], post-sequencing in silico quality assessment is strongly recommended for diagnosis and assurance purposes. Although some tools have been adopted or developed for quality control and analysis of ATAC-seq data in the past years (see Introduction and Additional file 2: Table S1), to the best of our knowledge, our *ATACseqQC* package provides the most comprehensive and integrated set of functionalities for both quality assessment and preprocessing of aligned ATAC-seq data for further downstream analysis. Besides most of the commonly adopted QA metrics such as fragment size distribution, mitochondrial read fraction, duplication rate, and aggregated read distribution along proximal promoters, *ATACseqQC* also provides several new functionalities such as gene-centric view of signal distribution, library complexity evaluation and sequencing depth analysis.

An appropriate fragment size distribution is generally a prerequisite for a successful ATAC-seq assay. However, this metric alone is not necessarily a sufficient condition for a library to be of good quality. Thus, in our package, simple analysis of size distribution is integrated with several additional diagnostic plots and analyses, such as aggregated TSS enrichment plots, footprint plots of tightly bound DNA-binding factors, and gene-level visualization of ATAC-seq signal. By applying these additional diagnostic tools, we can achieve greater confidence in library quality. For example, because promoter regions of active genes are in an open chromatin state and have a stereotypical pattern of ATAC-Seq mapping, we can use the TSS enrichment plot, in parallel to size distribution, to distinguish high quality libraries from low quality ones by visualizing nucleosome-free fragment density around TSS across the genome. An important observation from our application of *ATACseqQC* on previously published libraries is that an ATAC-seq library of an optimal or near-optimal distribution of fragment size may not necessarily have enough complexity or sequencing depth. Therefore, we implemented and integrated several new functions, such as *estimateLibComplexity* and *saturationPlot* into *ATACseqQC* (Table 1) that together provide a comprehensive assessment of ATAC-seq library quality.

## Conclusions

To aid the quality assessment of ATAC-seq experiments, we have developed the *ATACseqQC* package. This package can generate publication-quality diagnostic plots including fragment size distribution, nucleosome positioning pattern around TSSs, footprints of DNA-binding proteins of known binding motifs. In addition, the package has utilities for sequence depth analysis, library complexity evaluation, quality assessment on BAM files, and data preprocessing such as filtering alignments, shifting aligned reads, and separating reads into nucleosome-free and bound bins.

This package has facilitated the analysis of several in-house ATAC-seq experiments, including one recently published [45]. It will also help researchers to select high quality ATAC-seq datasets from public repositories such as GEO for re-analysis to avoid generating hypotheses or drawing conclusions from low-quality ATAC-seq experiments. In addition, this package could be incorporated into a pipeline for data centers such as GEO or ENCODE to evaluate each submitted ATAC-seq dataset before accepting and releasing the dataset for public consumption.

### Availability and requirements

Project name: ATACseqQC.

Project home page: https://bioconductor.org/packages/release/bioc/html/ATACseqQC.html.

Operating systems: Platform independent.

Programming language: R.

Other requirements: None.

License: GNU GPL.

Any restrictions to use by non-academics: None.

## Additional files

Additional file 1: Commands and scripts used for case studies. (TXT 19 kb)

Additional file 2: **Table S1.** Functional comparison between *ATACseqQC* and existing tools for ATAC-seq data QC analyses. (XLSX 14 kb)

Additional file 3: **Table S2.** Summary statistics of base quality of raw reads, mapping and filtering of read alignments. The four datasets chosen for detailed quality control are highlighted in bold. (XLSX 21 kb)

Additional file 4: **Figure S1.** Size distributions of sequenced fragments passing filtering criteria. NCBI SRA accession numbers for each ATACT-seq dataset are listed above each sub-fig. (A-**I**) are based on a study by Buenrostro et al. 2013; (J-O) are based on a study by Wijetunga et al. 2017; (P-S) are based on a study by Conrad et al. 2017; (T-Y) are based on an unpublished study by Vallés AJ and Izquierdo-Bouldstridge A. (PDF 572 kb)

Additional file 5: **Figure S2.** Read distribution along genomic regions containing housekeeping genes. (A) ACTB; (B) VCP; (C) REEP5; (D) RAB7A; and (E) VPS29. (PDF 289 kb)

Additional file 6: **Figure S3.** Diagnostic plots for subsampled datasets. Figs. A-D, E-H, I-L and M-P are based on 10%, 25%, 50% and 75% of randomly sampled reads from the post-filtered BAM file for dataset SRR891270. (A, E, I and M) fragment size distributions; (B, F, J and N) Heatmaps showing signals around TSSs; (C, G, K and O) distributions of averaged coverage; (D, H, L and P) aggregated CTCF footprints. (PDF 1197 kb)

## Abbreviations

ATAC-seq: Assays for Transposase-Accessible Chromatin using sequencing
BAM: Binary Alignment/Map
bp: base pair
CTCF: CCCTC-binding factor
DNase-seq: DNase I digestion of chromatin followed by high throughput sequencing
FAIRE-seq: Formaldehyde-Assisted Isolation of Regulatory Elements followed by high throughput sequencing
MNase-seq: Micrococcal Nuclease digestion of chromatin followed by high throughput sequencing
NCBI SRA: the National Center for Biotechnology Information Short Read Archive database
NRF: Non-redundant fraction
PBC1: PCR bottleneck coefficients 1
PBC2: PCR bottleneck coefficients 2
PCR: Polymerase Chain Reaction
PWM: Position Weight Matrix
SAM: Sequence Alignment/Map
TSS: Transcription Start Site
