# Supplementary figures and images for: ATACseqQC: a Bioconductor package for post-alignment quality assessment of ATAC-seq data
Source: BMC Genomics. 2018 Mar 1;19:169. doi: 10.1186/s12864-018-4559-3 (PMC5831847; doi:10.1186/s12864-018-4559-3)

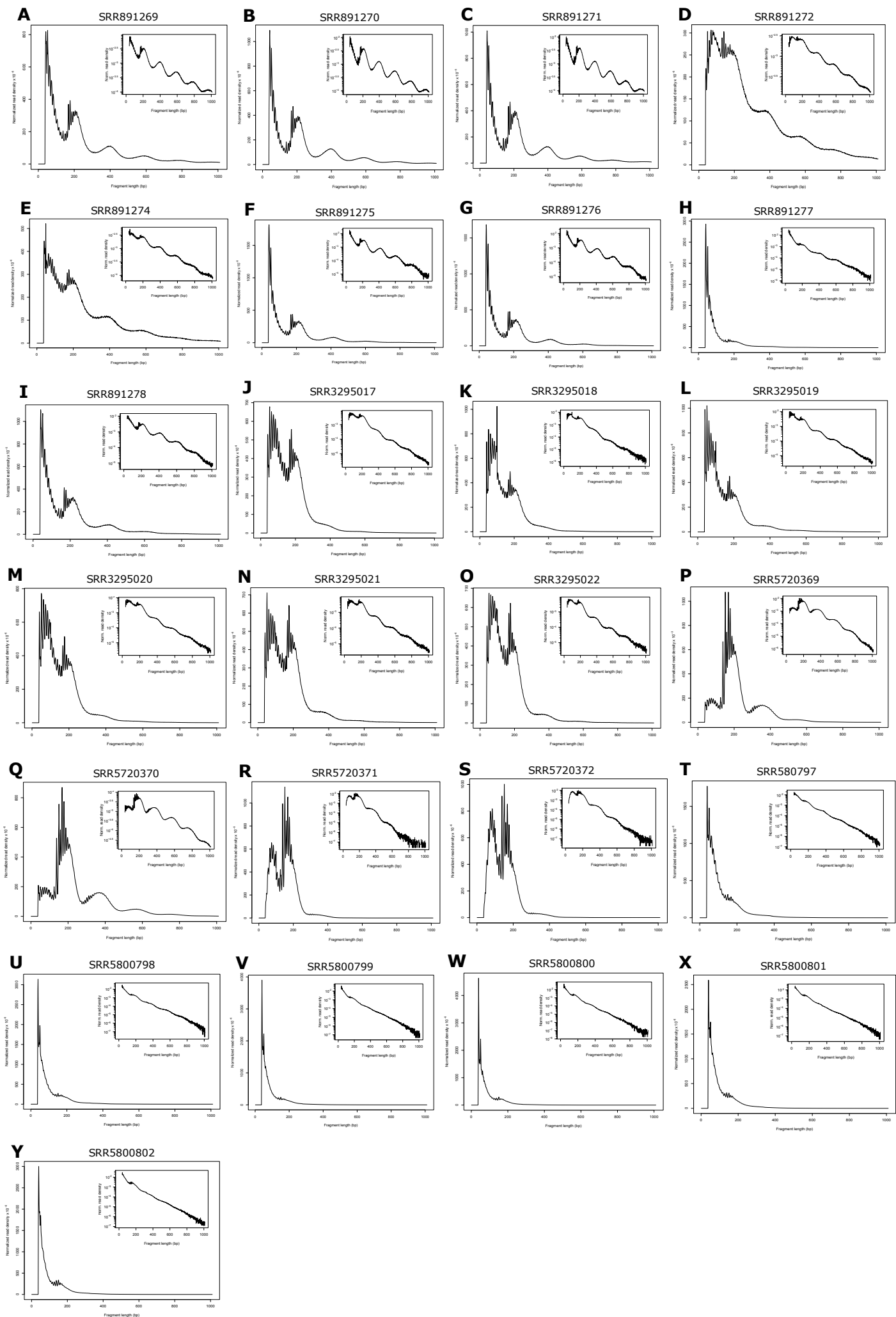

Supplement: Supplementary file 4 — Figure S1. Size distributions of sequenced fragments passing filtering criteria. NCBI SRA accession numbers for each ATACT-seq dataset are listed above each sub-fig. (A-I) are based on a study by Buenrostro et al. 2013; (J-O) are based on a study by Wijetunga et al. 2017; (P-S) are based on a study by Conrad et al. 2017; (T-Y) are based on an unpublished study by Vallés AJ and Izquierdo-Bouldstridge A. (PDF 572 kb) [file 12864_2018_4559_MOESM4_ESM.pdf]

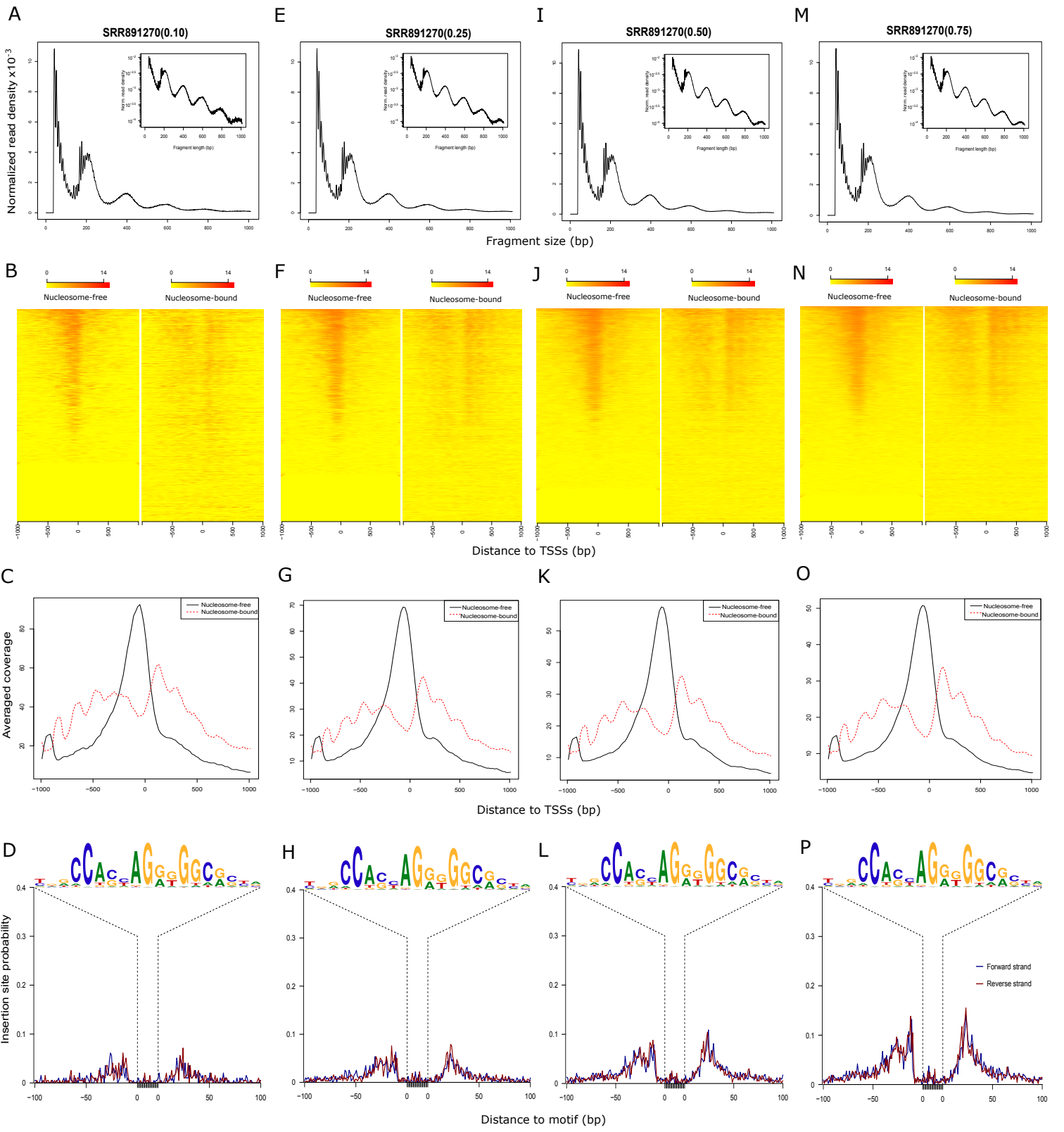

Supplement: Supplementary file 6 — Figure S3. Diagnostic plots for subsampled datasets. Figs. A-D, E-H, I-L and M-P are based on 10%, 25%, 50% and 75% of randomly sampled reads from the post-filtered BAM file for dataset SRR891270. (A, E, I and M) fragment size distributions; (B, F, J and N) Heatmaps showing signals around TSSs; (C, G, K and O) distributions of averaged coverage; (D, H, L and P) aggregated CTCF footprints. (PDF 1197 kb) [file 12864_2018_4559_MOESM6_ESM.pdf]
