# Supplementary material for: ATACseqQC: a Bioconductor package for post-alignment quality assessment of ATAC-seq data
Source: BMC Genomics. 2018 Mar 1;19:169. doi: 10.1186/s12864-018-4559-3 (PMC5831847; doi:10.1186/s12864-018-4559-3)
Supplement: Supplementary file 5 — Figure S2. Read distribution along genomic regions containing housekeeping genes. (A) ACTB; (B) VCP; (C) REEP5; (D) RAB7A; and (E) VPS29. (PDF 289 kb) [file 12864_2018_4559_MOESM5_ESM.pdf]

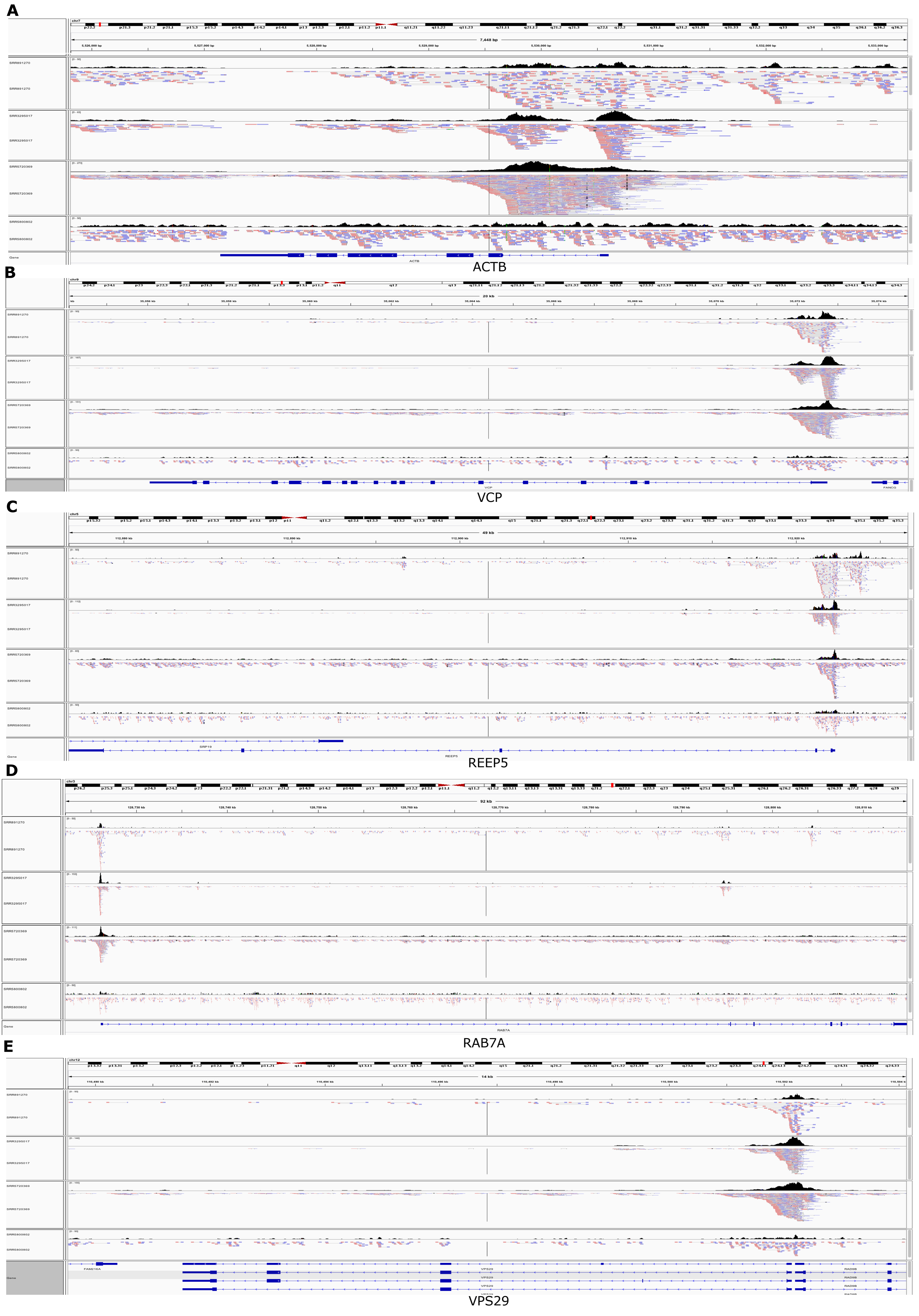
A
chr7
7,448 bp
SRR891270
SRR3295017
SRR5720369
SRR5800802
Gene
ACTB
B
chr9
20 kb
VCP
FANCG
C
chr5
49 kb
SRP19
REEP5
D
chr3
92 kb
RAB7A
E
chr12
14 kb
FAM216A
VPS29
RAD9B
